# Influence of MLS laser radiation on erythrocyte membrane fluidity and secondary structure of human serum albumin

**DOI:** 10.1007/s11010-013-1917-y

**Published:** 2013-12-20

**Authors:** Kamila Pasternak, Olga Nowacka, Dominika Wróbel, Ireneusz Pieszyński, Maria Bryszewska, Jolanta Kujawa

**Affiliations:** 1 Clinic of Medical Rehabilitation, Medical University of Lodz, 75 Drewnowska Str, 91-002 Lodz, Poland; 2 Department of General Biophysics, Faculty of Biology and Environmental Protection, University of Lodz, 141/143 Pomorska Str, 90-236 Lodz, Poland

**Keywords:** Erythrocyte membrane, Human serum albumin, Laser therapy, MLS M1 system

## Abstract

The biostimulating activity of low level laser radiation of various wavelengths and energy doses is widely documented in the literature, but the mechanisms of the intracellular reactions involved are not precisely known. The aim of this paper is to evaluate the influence of low level laser radiation from an multiwave locked system (MLS) of two wavelengths (wavelength **=** 808 nm in continuous emission and 905 nm in pulsed emission) on the human erythrocyte membrane and on the secondary structure of human serum albumin (HSA). Human erythrocytes membranes and HSA were irradiated with laser light of low intensity with surface energy density ranging from 0.46 to 4.9 J cm^−2^ and surface energy power density 195 mW cm^−2^ (1,000 Hz) and 230 mW cm^−2^ (2,000 Hz). Structural and functional changes in the erythrocyte membrane were characterized by its fluidity, while changes in the protein were monitored by its secondary structure. Dose-dependent changes in erythrocyte membrane fluidity were induced by near-infrared laser radiation. Slight changes in the secondary structure of HSA were also noted. MLS laser radiation influences the structure and function of the human erythrocyte membrane resulting in a change in fluidity.

## Introduction

Laser therapy is a physiotherapeutic method widely used in rehabilitation [1]. It is a noninvasive method of symptomatic treatment effective in pain reduction [2], and a causative treatment for which anti-inflammatory and regeneration stimulating characteristics have been proven [3–5]. Laser biostimulation causes changes in cellular metabolism reflected in increased intensity of ATP synthesis, increased protein synthesis (DNA and RNA), increased cellular proliferation, increased enzyme activity (e.g. ATPase), increased number of mitochondria, and increased membrane potential. Tissue oxygen supply is improved through increased blood perfusion and accelerated hemoglobin dissociation [6–8]. However, the mechanism by which living material reacts to low level laser radiation on the cellular or tissue level has not been characterized. Low level laser therapy still raises controversy in spite of numerous reports of in vitro research, animal trials, and randomized controlled clinical trials confirming its positive effects [9, 10]. Moreover, some publications fail to confirm a significant effect of laser therapy [11]. Laser biostimulation commonly uses the emission of only one wave in a continuous or pulse regime. Progress in biomedical applications of laser radiation is triggered by constant research into new types of laser devices. The multiwave locked system (MLS) M1 system emits radiation at two wavelengths (*λ* = 808 nm in continuous and *λ* **=** 905 nm in pulse emission). To date, this type of synchronized radiation has scarcely been used, though some authors claim it has better therapeutic effects than traditional laser therapy [12, 13]. Few publications have described the therapeutic effectiveness of synchronized laser radiation on cellular structures and the human body and its differences from traditional forms of radiation therapy. Clinical tests have not demonstrated the advantage of synchronized laser radiation over traditional laser therapy unambiguously [14]. The development of optoelectronics has enabled the structures of molecules to be observed and examined. Spectropolarimetry is widely used as it allows the secondary structure of human serum albumin (HSA) to be evaluated and its changes in response to a wide range of factors to be characterized [15–17].

## Objectives

The aim of this paper is to evaluate the influence of MLS laser radiation on human erythrocyte membrane fluidity. HSA was used as a model for evaluating the secondary structure changes in proteins. The results of applying the synchronized MLS system radiation of two different wavelengths were analyzed in comparison with traditional laser radiation using a single wavelength (*λ* = 810 nm).

## Materials and methods

The research material comprised human erythrocyte membranes and HSA irradiated by the MLS M1 laser system (*λ* = 808 nm in continuous and *λ* = 905 nm in pulsed emission) with surface energy density ranging from 0.46 to 4.9 J cm^2^. The density of surface energy power in pulsed emission reached 195 (1,000 Hz) and 230 mW cm^−2^ (2,000 Hz). Laser radiation energy doses were: 0, 1.5, 3, 6, 9, 12, and 15 J.

### Erythrocyte membrane isolation

Blood was divided into two centrifuge tubes and centrifuged at 400×*g*, 4 °C for 10 min. The plasma and leukocytes were removed. The red blood cell suspension was washed three times with 0.9 % NaCl. Next, the red blood cell pellet was divided into 3–4 ml test tubes. Each portion was supplemented with five volumes of chilled buffer (20 mM of Tris–HCl, pH 7.4, 1 mM EDTA, 0.1 g/l PMSF, pH 7.4) and centrifuged at 19,000×*g* for 5 min. The supernatant was removed. The process was repeated until milky-white membranes and transparent supernatant were obtained. The protein concentration was measured by Lowry’s method [18].

### Erythrocyte membrane fluidity changes

A membrane suspension containing 50 μg of protein/ml in PBS was prepared. The fluorescent labels TMA-DPH or DPH were added to the sample at 1 μM. The labeled sample was incubated for 15 min. Each 2 ml aliquot of membrane suspension was irradiated. Fluidity was measured directly before and after irradiation and repeated six times for each sample. A Perkin-Elmer LS-50B spectrofluorimeter (Great Britain) was used for those measurements. The excitation wavelength was *λ* = 348 nm, and the emission wavelength *λ* = 426 nm. Excitation and emission slit widths were kept constant at 8.0 nm. GF = 1.46. Fluorescence anisotropy coefficients (*r*) were calculated using Jablonski’s equation:

1 $$r = \frac{{I_{\text{vv}} - GI_{\text{vh}} }}{{I_{\text{vv}} + 2GI_{\text{vh}} }},$$

where *I*_*vv*_ and *I*_*vh*_ are the vertical and horizontal fluorescence intensities, respectively, to the vertical polarization of the excitation light beam. The factor *G* = *I*_*hv*_ */I*_*hh*_ (grating correction factor) corrects the polarizing effects of the monochromator.

### Changes in HSA secondary structure measured by circular dichroism

The HSA was dissolved in PBS at 0.332 mg ml^−1^. The baseline was drawn using PBS. Next, the spectra of non-irradiated and irradiated HSA samples were measured. For this purpose, the sample was put into a 0.5 mm optical path quartz cuvette and spectra were taken from five independent samples in a circular dichroism **s**pectropolarimeter (JASCO J-815,GB) at 180–260 nm at 37 °C, scanning velocity 50 nm/min.

### Statistical analysis

Data for the six values obtained for each sample are presented as mean ± SEM. One way, ANOVA was used to compare the treated with the control group. Fisher’s LSD test and Student’s *t* test were used to determine statistically significant differences in multiple and two samples comparisons, respectively. Normal distribution parameters were checked using the Shapiro–Wilk test. The level of statistical significance was *α* = 0.05. Other analyses were performed using STATGRAPHICS Centurion XVI ver.16.1.18.

## Results

### The effect of laser light on erythrocyte membrane fluidity

Fluorescence anisotropy coefficients (r) were calculated using Eq. (1) and were used to evaluate the effect of MLS M1 laser radiation on erythrocyte membrane fluidity. Frequencies of 1,000 and 2,000 Hz were applied. Fluorescence anisotropy was measured for two fluorescent labels, DPH and TMA-DPH, located in different parts of the membrane.

### Changes in fluidity of hydrophobic region of erythrocyte membrane

**Fig. 1:**
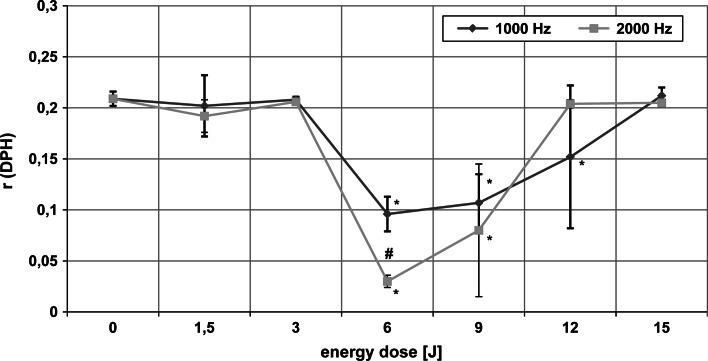
Dependence of fluorescence anisotropy coefficient (*r*) of DPH in erythrocyte membranes on the dose of MLS laser radiation at 1,000 and 2,000 Hz. *Asterick* statistically significant differences from 0 J dose. *Hash* statistically significant difference between the two frequencies at a fixed energy dose

The mean values of the fluorescence anisotropy coefficient (*r*) for the DPH dye located in the hydrophobic region of the erythrocyte membrane, for various energy doses and the two frequencies of laser radiation, are presented in Fig. 1. ANOVA revealed a significant dose effect of laser light for both emission pulse frequencies used in the experiment (*p* < 0.01). Fisher’s LSD test demonstrated that at 1,000 Hz, energy doses of 6, 9, and 12 J caused a statistically significant increase in membrane fluidity in the hydrophobic region of the membrane compared to the control sample (0 J). However, for 2,000 Hz, a similar effect was obtained for energy doses of 6 and 9 J only. There were significant differences between the fluidity changes induced by the two frequencies at the 6 J dose (Student’s *t* test, *p* < 0.05). For the remaining doses these differences were not statistically significant.

### Fluidity changes in the external monolayer of the erythrocyte membrane

**Fig. 2:**
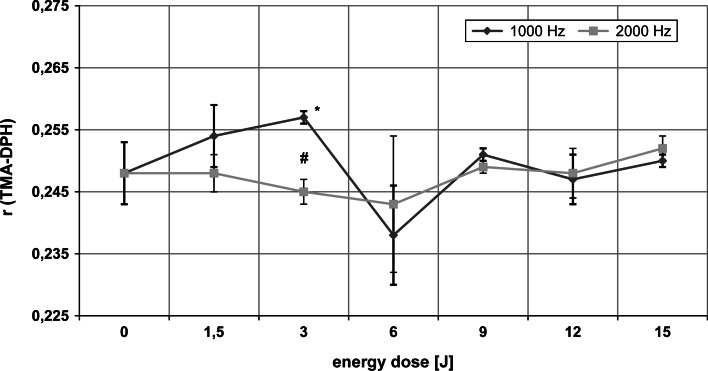
Dependence of fluorescence anisotropy coefficient (*r*) of TMA-DPH in the membranes of erythrocytes on MLS laser radiation doses at 1,000 and 2,000 Hz. *Asterick* statistically significant differences from 0 J dose. *Hash* statistically significant difference between the two frequencies at a fixed energy dose

Laser radiation affected the fluidity of the external region of the membrane significantly only for 1,000 Hz (ANOVA, *p* < 0.01). For the 3 J dose only, the membrane fluidity was significantly lower than in the control (0 J). Statistically significant differences in fluidity were found at both laser frequencies only for this energy dose (3 J) (Student’s *t* test, *p* < 0.01) Fig. 2.

### Changes in the secondary structure of HSA

**Fig. 3:**
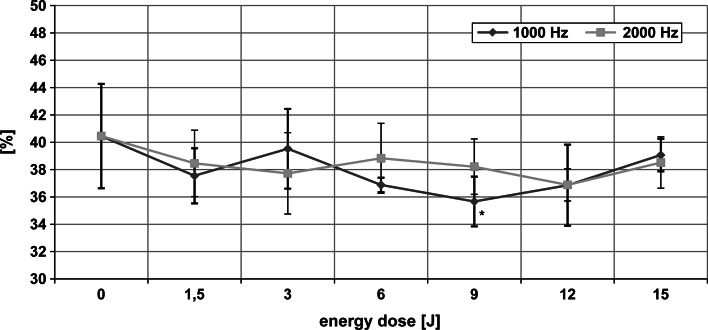
The content of α-helix in HSA after irradiation with laser light. *Asterick* Statistically significant differences from 0 J dose

**Fig. 4:**
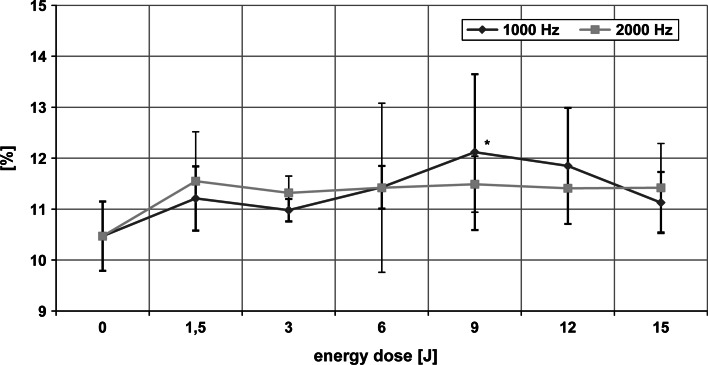
The content of beta structures in HSA upon laser light irradiation. *Asterick* Statistically significant differences from 0 J dose

**Fig. 5:**
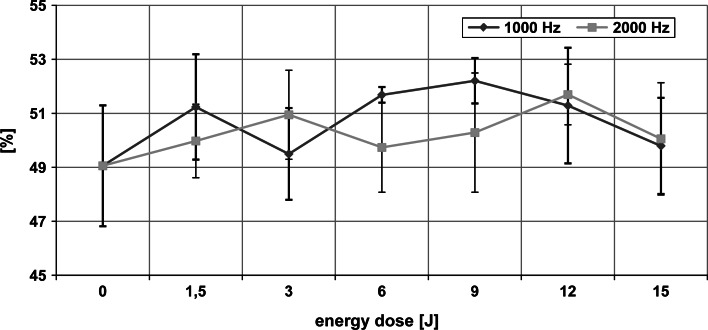
The content of random structures in HSA upon laser light irradiation

The CD spectrum of HSA subjected to laser radiation allowed the effect of the light doses and frequencies used on the secondary structure of the protein to be assessed. Changes in the contents of various structures in HSA on irradiation are illustrated in Figs. 3, 4, and 5. The percentage of α-helix was lower than in the control sample after a 9 J dose at 1,000 Hz frequency (ANOVA, *p* < 0.001). Radiation with the same characteristics increased the beta structure content of HSA (ANOVA, *p* < 0.01). The content of random structures remained the same as in the non-irradiated sample. Exposure of HSA to laser radiation at 2,000 Hz over the entire range of applied energy doses caused no statistically significant changes in any secondary structures.

There were no statistically significant differences in the contents of the various structures in HSA between the same doses of radiation at different frequencies (1,000 and 2,000 Hz) (Figs. 3 and 4). Analysis of results showed a tendency for alpha helix to decrease and beta structures and random structures to increase over the energy range 6–12 J at 1,000 Hz. The opposite effect was observed at 2,000 Hz.

## Discussion

A broad spectrum of changes can be induced in cells and biological membranes by laser radiation. Much research has revealed the influence of laser radiation on the activities of membrane enzymes such as acetylocholinoesterase (AChE) or (Na^+^, K^+^) ATPase [19–22].

In this study, we demonstrated the effect of laser radiation on the fluidity of hydrophobic and hydrophilic regions of human erythrocyte membranes. The biological effect of radiation differed between the two regions. The changes in membrane fluidity occurred in the energy range 6–12 J at 1,000 Hz and 6–9 J at 2,000 Hz in the hydrophobic region of the membrane, but the above frequencies exerted biological effects only with 6 J energy. The fluidity of the surface layer of the erythrocyte membranes in the irradiated samples was changed for only one energy dose, 3 J at 1,000 Hz. The biological effects of the two frequencies differed at this energy dose.

Comparing fluidity changes in the two membrane regions, a ‘shift’ in the biological effect towards higher energy doses is apparent for both laser radiation frequencies. It is plausible that the results obtained in this work are mainly attributable to the radiation wavelength, i.e. the depth of penetration.

The erythrocyte membrane contains not only lipids, but also other elements such as proteins and glycoproteins on the surface. Another difference between the two membrane regions is the content of specific molecules that absorb laser radiation to different extents. This fundamental asymmetry in structure could be responsible for the differences in absorption and scattering of radiation by individual membrane constituents.

It was also noted that doses that did not cause changes in the fluidity of the surface layer exerted biological effects in the hydrophobic membrane region. This could be the result of applying too strong a stimulus to the surface layer—according to the Arndt-Schultz law [23]—or of the depth of penetration dependent on the laser radiation wavelength.

It may be assumed that some of the radiations were absorbed and the amount of energy that reached the internal region of the membrane can be described as a biostimulating agent.

Data in the literature reveal a statistically significant influence of laser radiation on human erythrocyte membrane fluidity [20, 24].

The increase in human erythrocyte membrane fluidity was statistically significant and dependent on the dose of applied radiation characterized by following values: wavelength 810 nm, power surface density 125 mW cm^−2^, energy doses: 3, 6, 9, 12, 15, and 20 J (with respective power surface densities 3.75, 7.5, 11.25, 15, 18.75, and 25 J cm^−2^).

In research cited above, the label 12-AS was used as well as DPH and TMA-DPH. 12-AS enables changes in fluidity at the depth of 12th carbon atom in the membrane phospholipid fatty acid chains to be observed. Fluidity measurements using the fluorescent label 12-AS revealed a statistically significant increase in rigidity of the erythrocyte membrane for doses of 6, 12, and 15 J, while for doses of 9 and 20 J there was a statistically significant fluidization of the membrane.

The single continuous wavelength radiation used in this research affected erythrocyte membrane fluidity more strongly than the MLS radiation system. This could be attributed to the wavelengths used or different methods of administration of the laser radiation.

Kujawa et al. [20] applied continuous radiation, while the MLS system emits synchronized continuous and pulsed radiation. Pulsed radiation has proven more effective. The MLS radiation effect was dependent on the energy doses used (surface power density 0.46–4.9 J cm^−2^) and was visibly lower than with continuously applied doses (surface energy density of radiation used in cited research was 3.75–25 J cm^−2^). It is worth emphasizing that the surface energy density was higher in the research conducted by Kujawa et al.

Similar research was conducted by Bryszewska and co-workers [22], who irradiated erythrocyte membranes with low level visible radiation (*λ* = 670 nm, power 7 mW, dose 5 J). In this research, the TMA-DPH fluorescent label was used. The results confirmed the influence of laser radiation on erythrocyte membrane viscosity. For the radiation dose of 5 J, the fluidity in the polar region of the erythrocyte membrane was significantly reduced. It was concluded that these changes in fluidity were caused by structural modifications in the lipid bilayer and lipid–protein interactions.

The effect of laser radiation on protein elements of the plasma membrane is complex. Membrane ATPase and AChE activities were affected by laser radiation, but the exact mechanism was not fully explained. This mechanism could be of two types: the laser radiation could affect the structure of the enzyme, resulting in activation or inactivation, or it could affect the enzyme environment leading to an indirect effect on activity.

HSA constitutes ~60 % of the plasma protein mass and is important in regulation of oncotic pressure, blood buffering, and binding and transporting many biological molecules such as hormones, drugs, and CO_2_.

Reduced oncotic pressure can cause edema. Evaluation of the changes in HSA structure caused by radiation can help to establish the precise dosage of radiation required to achieve a positive therapeutic effect. The results of this research could suggest that MLS laser radiation at 1,000 Hz and energy dose 9 J significantly reduces the α-helix content of HSA and, conversely, increases the beta structure content. There were no significant changes in random structures.

For laser irradiation at 2,000 Hz, no significant changes in HSA structure content was obtained, irrespective of energy dosage.

Analysis of the results reveals that doses of 6–12 J, 1,000 Hz, reduce the α-helix content of HSA and increase the content of other structures. The opposite effect is noted for 2,000 Hz. It is worth emphasizing that the increase of α-helix and decrease in other structures was found for the 3 J dose at 1,000 Hz, contrary to that at 2,000 Hz. However, those changes were not statistically significant. The structural changes in HSA were not induced by thermal effects because of the low level of energy radiation used in this research.

HSA structural changes resulting from thermal factors have been described by Rezaei-Tavirani et al. [25]. Using circular dichroism spectroscopy they proved that a rise in temperature from 25 to 55 °C causes reversible conformational changes in HSA. The thermal effect is negligible for low level energy laser radiation.

Comparing the energy doses applied at different frequencies, it can be noted that doses ranging from 1.5 to 3 J and from 6 to 12 J cause opposite biological effects in both the HSA and the surface region of the erythrocyte membrane.

Conducting research with lower energy dose intervals might explain whether the observed changes in the values monitored are of statistical and clinical significance.

## Conclusions

MLS laser radiation influences the structure and function of the human erythrocyte membrane resulting in a change in fluidity.
